# Comparison of sedative effects of oral midazolam/chloral hydrate and midazolam/promethazine in pediatric dentistry

**DOI:** 10.15171/joddd.2018.034

**Published:** 2018-09-18

**Authors:** Majid Mehran, Ghassem Ansari, Mojtaba Vahid Golpayegani, Shahnaz Shayeghi, Leila Shafiei

**Affiliations:** ^1^Department of Pediatric, Faculty of Dentistry, Shahed University of Medical Science, Tehran, Iran; ^2^Department of Pediatric, Faculty of Dentistry, Shahid Beheshti University of Medical Science, Tehran, Iran; ^3^Department of Anesthesiology, Faculty of Dentistry, Shahid Beheshti University of Medical Science, Tehran, Iran; ^4^Private Practice, Kerman, Iran

**Keywords:** Child, chloral hydrate, dental sedation, midazolam, promethazine

## Abstract

***Background.*** The aim of this investigation was to compare the sedative effects of oral midazolam/chloral hydrate and midazolam/promethazine combinations on fearful children needing dental treatment.

***Methods.*** This crossover double-blind clinical trial was conducted on 30 children aged 2‒6 years, who had at least two similar teeth needing pulp treatment. Standard vital signs were recorded before and after premedication. Wilson sedation scale was used to judge the level of sedation. Cases were divided into two groups based on the sequence of medication received. This was to overcome the sequence effect. Group I received oral midazolam (0.4 mg/kg/chloral hydrate (50 mg/kg) at the first visit while they received midazolam (0.4 mg/kg)/promethazine (5 mg/kg) in their second visit. Group II received the premedication in the opposite sequence. The operator and child were blinded to the medication administered. Sedative efficacy of the two combinations were assessed and judged by two independent pediatric dentists based on the Wilson scale. Data were analyzed with ANOVA and paired t-test.

***Results.*** Only 10% of children who received chloral hydrate with midazolam exhibited high improvement in their behavior while 53% showed reasonable positive changes and 12% had no change or even deterioration of behavior. The difference between the effect of the two combination drugs was statistically significant (P<0.05) in favor of the chloral hydrate group.

***Conclusion.*** The results showed a significant difference in the sedation level induced between the two groups. Midazolam/chloral hydrate combination more effectively improved the co-operation for dental treatment.

## Introduction

Dental visits have long been a concern and for many as a major challenge at young age. Fear and anxiety could lead to serious avoidance of treatment when it is in its critical stages. This will, in turn, lead to a more complicated status, which sometimes compromises the chance for saving the teeth. Many dental professionals seldom see children due to the pretext of being unable to cope with their treatment, usually ending in an uncompleted dental procedure.^1,2^ Child dental anxiety is widely acknowledged as the main source of behavior problems in the dental office.^3^ These cases are best treated by pharmacological assistance using certain sedative agents.^4^ Patients who are judged negative in behavioral scale can be clinically seen only when subjected to a pharmacological preparation.^5,6^ Conscious sedation is considered as one of the most convenient yet safe currently available premedications for delivering dental care in children, among which oral sedation has the highest acceptance rate by patients.^6^ Simplicity of the sedative agents used will provide degrees of sedation needed to reduce child’s interfering movement while receiving treatment with oral route being on top.,^7^ Of course, this is not necessarily the case in those very young fearful children.^6^ Varying degrees of side effects, including overdose, idiosyncrasy and allergy could also be seen with the administration of these drugs.^7^ While the oral route is associated with minimal sedative reactions when compared to other routes, its gentle effect on patient allows easy and smooth recovery after treatment with less complications. Late-onset effect is considered as a disadvantage to oral sedation along with unpredictable absorption rate and titration limitations. Appropriate case selection is the key to having the best and reasonably effective oral sedation results. In most instances, the initial sedative signs are seen at and around half hour of administration wken the pick plasma level of the drug is reached to obtain its anxiolytic effects.^4^

Oral sedative drugs usually include anxiolytics, sedatives, barbiturates, narcotics and antihistamines.^8-10^ The use of inhalation sedation is routine when associated with other routes, including oral sedation.^8,11,12^ Oral midazolam has been used as a short- and fast-acting benzodiazepine prior to GA and several other medical diagnostic approaches for many years.^4, 8,12-14^ Promethazine is a known antihistaminic drug with degrees of sedative effect, particularly when associated with other agents. It is well absorbed in the gastrointestinal system with its effect starting within the first 20 minutes of oral administration. This effect will diminish after a period of 4‒6 hours. Promethazine is also used to control postoperative nausea, vomiting and apprehension.^7^ Most sedative drugs reach their highest level of effect after 30 minutes of their administration.^4-8^ As most of the sedative agents have the potential to take the patient to deep states, it is always desired to look for a more moderate sedative agent to enable a lighter sedation mode with closer monitoring to better control the patient while sedated. This is thought to be provided with the use of antihistamines along with low doses of benzodiazepines. Titration problem in oral sedation is a pitfall and obstacle to obtaining an efficient clinical state while parenteral routes of the same drugs provide lasting effects of sedation (3‒4 hours) for efficient treatment; however, the risks of airway depression increases, too. Besides, deeper sedation would trigger late discharge due to late sedative effects, highlighting the benefits of lighter oral sedation.^9-14^

On the other hand, several earlier studies have looked at the combined sedative effects of various drugs used alongside midazolam. This is believed to have a synergic effect on sedation and therefore a lower dose is needed, which would provide efficient sedation level suitable for delivering dental treatment.^1,4,8^ As an example, ketamine and midazolam have already been used successfully with lower doses compared to their single use for dental treatment of young fearful children.^4,8,12^ Potential side effects of ketamine and chloral hydrate when used orally include nystagmus, hallucination and hypersalivation.^13,15^ A few different cocktails of sedative agents have been tested successfully for children, among which less attention has been paid to the use of promethazine or chloral hydrate along with midazolam, for dental procedures. The use of promethazine/midazolam allows lower doses to be used with almost the same effect comparable to two sedatives of chloral hydrate/midazolam. This can be administered in a safe way in clinics.

This study was designed to evaluate and compare the sedative effects of two combinations of oral drug regimens, including chloral hydrate/midazolam and promethazine/midazolam in a group of uncooperative child dental patients aged 2‒6 years.

## Materials and Methods

This crossover double-blind clinical trial was conducted on 30 children aged 2‒6 years, who had at least two similar teeth needing pulp treatment. Case selection was performed based on their attendance and in a sequential manner (simple sampling). Cases that were judged definitely negative in Frankl behavioral scale were included, who were also classified as ASA I. Cases were selected from referrals to outpatient clinic in a Children’s Hospital, Tehran, for arrangement to receive dental treatments under general anesthesia or sedation. The combination of drugs was administered at the start of each dental session by an anesthesiologist with dental operator and patient blinded to the drugs combination during the visit.

Each patient was assigned to receive two dental visits with two similar teeth needing relatively similar dental treatment in one jaw. Each case would therefore attend two appointments for two different combination drugs as the test and control. To avoid the sequence effect, the patients were randomly allocated to two separate groups in order to receive the test or control combination drugs in a specific order. Inclusion criteria were: cases that were confirmed as having no signs of any systemic problem, cold or other nasal or respiratory infections, no enlarged tonsils, no problem with neck movements and no oversized tongue. All the required information and instructions were given to parents in writing preoperatively, including the consent form for the child’s participation in this trial. Ethical approval was obtained from the Ethics Committee of Shahid Beheshti University of Medical Sciences prior to the commencement of the investigation. A 6-hour period of fasting (NPO) was instructed, preoperatively. The drugs were administered in the first or second visits of each patient in a random manner while each patient served as self control. A combination of 0.4 mg/kg of midazolam (5 mg/mL vials, Tehran Chemicals, Tehran, Iran) mixed with sweetened water was administered along with 5 mg/kg of chloral hydrate elixir (5 mg/mL, Daroupakhsh, Iran) in one session while 5 mg/kg of midazolam was administered along with 5 mg/kg of promethazine orally in the next visit. The dental procedure was started after a 30-minute waiting period in order to achieve the best possible oral effects of sedation.^4,1,6^

The second combination consisted of 0.4 mg/kg of midazolam with 5 mg/kg of promethazine (5 mg/mL Elexir, Sina Daru, Iran). All the vital signs were constantly monitored, including blood pressure, oxygen saturation and pulse rate, starting at baseline followed by recording every 15 minutes up to the discharge point, using a vital sign monitor (Saadat, Iran). A pediatric dentist performed all the treatment procedures while child’s behavior rating was conducted by another independent pediatric dentist. Wilson’s Sedation scoring system was used to record child’s behavior pre-, intra- and post-operatively.^12,14^ Blood pressure (BP) and oxygen saturation (OS) were measured and recorded as the two main components of the child’s vital status.^13^ In case of refusal due to lack of sufficient sedation the case was excluded from the study and treatment was completed using deeper sedation. The patients were discharged after full recovery from sedative signs as judged by the anesthesiologist in charge. The parents were encouraged to report all the potential postoperative complications. Data were statistically analyzed using ANOVA for vital sign differences, Friedman's test for sedation differences and paired t-test for differences before and after sedation in each group. Wilcoxon’s signed rank test was also used for inter-group comparisons.^14^

## Results

The mean age of the subjects was 43.5 months (±10.9) with 48.4% of them being male. There was a greater improvement in cooperation level of those receiving midazolam/chloral hydrate combination when compared to those receiving midazolam/promethazine combination (P<0.05). The least value for oxygen saturation level was 94% among the entire population of the study with no statistically significant difference between 6 readings of the groups (P<0.001). Pulse rate changes clearly changed from pre-operation to the time of treatment and after sedation when chloral hydrate was administered compared to the promethazine group with a significant difference between groups using paired t-test (P<0.002). Wilson behavioral evaluation scale showed a considerable rise in cooperation level of children treated with chloral hydrate (48.4%) (Table 2). However, no significant difference was found between the cooperation rate of the two groups after the sedation was induced (P=0.66). Sedation scale of Wilson showed better cooperation after sedation was induced compared to that before sedation (Table 3). However, the depth and length of this sedative status was more evident in the chloral hydrate/midazolam group compared to the promethazine/midazolam group, which was statistically significant (P<0.001). The differences in cooperation level of children of the two groups was not significant between the recorded times: before and after the treatment (P=0.025 for CH and P=0.763 for PM).

**Table 1:** Wilson sedation score

**Score**	**Signs**
**1**	**Fully aware and oriented**
**2**	**Drowsy**
**3**	**Eyes closed but responsive to command**
**4**	**Eyes closed but responsive to mild physical stimulation (earlobe tug)**
**5**	**Eyes closed but unresponsive to mild physical stimulation**

**Table 2:** Frequencies of the Wilson’s behavior score before and after dental treatments in each sedation group

			**Wilson**	**behavior**	**Score**			
**Intervention**	**Recoded time**	**Interrupting No (%)**	**Bad No (%)**	**Not bad No (%)**	**Good No (%)**	**Excellent No (%)**	**Mean rate in Friedman test**	**P-value**
	Before Dental	0	3 (9.7)	12 (38.7)	11 (35.5)	5 (16.1)	3.10	
**Chloral hydrate**	During Dental	5 (16.1)	0	7 (22.6)	4 (12.9)	15 (48.4)	4.16	P>0.05
	After dental	5 (16.1)	3 (9.7)	4 (12.9)	4 (12.9)	15 (48.4)	3.90	
	Before Dental	0	0	18 (58.1)	13 (41.9)	0	3.26	
**Promethazine**	During Dental	5 (16.1)	3 (9.7)	6 (19.4)	11 (35.5)	6 (19.4)	3.19	P>0.05
	After dental	0	10 (32.3)	4 (12.9)	11 (35.5)	6 (19.4)	3.39	

**Table 3:** Frequencies of the Wilson’s sedation score before and after dental treatments in each sedation group

		**Wilson**	**Sedation Score**		
**Intervention**	**Recoded time**	**Anxious and awake** **No. (%)**	**Sleepy No. (%)**	**Mean rate in Friedman test**	**P-value**
**Chloral hydrate**	Before treatment	29 (93.5)	2 (6.5)	2.02	
	During treatment	0	30 (100)	4.82	P>0.05
	After treatment	9 (29.0)	22 (71.0)	3.95	
**Promethazine**	Before treatment	29 (93.5)	2 (6.5)	2.02	
	During treatment	15 (16.1)	26 (83.9)	4.34	P>0.05
	After treatment	10 (32.3)	21 (67.7)	3.85	

## Discussion

There are still many children who suffer from severe dental problems due to their extreme anxiety and fear. Many pediatric dentists and even dental schools still use routine office behavioral control techniques, including physical restraints as their only available resource. More recent intensive research indicates that premedication is a useful tool to shape the child’s behavior in the dental clinic. Oral route of sedation has been widely agreed on as the most convenient yet effective way to overcome fear and anxiety in young fearful children. The taste of medication remains a challenge for the operator, which can be resolved when it is added to sweetened water or a juice. Damle et al^4^ (2008) reported midazolam as an agent capable of inducing sufficient level of sedation when prescribed as the only medication.

Chloral hydrate has been the drug of choice for many years based on its highly desired efficacy; however, it has potential risks which make its daily dental use compromised.^17^ The results of this study showed the benefits of the use of cocktail drugs in favor of reducing the sedative dose required to achieve the optimum result. The use of an antihistaminic drug such as promethazine reduces the risk of postoperative nausea and vomiting as an expected consequence of sedation. No significant differences were seen between the level of blood oxygen saturation before and after sedation was induced in both groups (P=0.119). Similar findings were reported by Vahid et al^18^ (2012) with ketamine/midazolam. Chawdhury and Vargas^19^ also reported that chloral hydrate and meperidine did not show any significant differences in their sedative effect. Sheroan et al^15^ (2006) reported a slight change in blood oxygen saturation level when meperidine/chloral hydrate combination was administered. Any drop in blood oxygen saturation was treated seriously when a patient was sedated unless it was within the normal range of 95‒100%.^20^ Clearly the drug type and its dose have a direct impact on oxygen saturation rate and its reduction in certain cases can lead to airway obstruction.^21^ Dallman et al^22^ (2001) did not report any major oxygen saturation change following the use of midazolam with and without chloral hydrate and promethazine.^22^ Heart rate changes were noticeable to some extent in both groups of this study at different stages, indicating the direct effect of the drugs used.

Since young children are highly vulnerable to the medication used for various purposes, including sedation, it is important to make every attempt to bring the risks to the minimum level possible. This is valuable if the effectiveness of the drugs used could provide the optimum level of cooperation for the desired dental procedure to be performed in a safe mode. The combination drug use consists of a strategy in which studies, including the current one, confirm that lower doses of the known sedative agents can be administered safely in children while achieving maximum required sedation level to satisfactorily carry out the procedure. Evaluation of the potential effect of promethazine along with midazolam revealed no significant differences when stronger chloral hydrate/midazolam combinations were used, which was expected to act more effectively (P>0.05).

It appears that with the sedative effect of midazolam used in both groups there seems to be a good capacity for the use of a more conservative promethazine agent over more controversial chloral hydrate in pediatric dental patients in order to avoid untoward complications when cocktail sedation agents are being used. Interestingly, chloral hydrate use has declined in recent years based on its side effects, supporting the outcome of this investigation.^23^ Oral sedation combinations clearly produce various levels of sedation and it is the direct responsibility of the operating clinician to use the best method and regimen in a carefully selected manner.^24^

## Conclusion

1. There was no significant difference between the sedation levels induced by chloral hydrate/midazolam and promethazine/midazolam.
2. Child’s postoperative problems were much less in the promethazine group.
3. There were no significant differences between the two groups in their vital signs.
4. No sequence effect was seen following the use of one combination over the other.

## Acknowledgments

Authors would like to express their appreciation for support and help from Mr Moatamedi, Ms Oghbaei, Patients and their parents and Dental School Research Institute at Shahid Beheshti Medical University for their financial and scientific support.

## Acknowledgments

Authors would like to express their appreciation for support and help from Mr Moatamedi, Ms Oghbaei, Patients and their parents and Dental School Research Institute at Shahid Beheshti Medical University for their financial and scientific support.

## Authors’ contributions

MM, GhA, ShSh and MVG were involved in designing the study and collection of data. The rest of the authors were involved in drafting and final approval of the manuscript.

## Funding

The study was financially supported by the Research Center of Shahid Beheshti Medical University.

## Competing interests

The authors declare no competing interests with regards to the authorship and/or publication of this article.

## Ethics approval

The ethical clearance was obtained from the institutional ethics committee. All the patients’ parents involved in this study gave written consent for participation and publication of the research data.
